# Rehydration Capacities and Rates for Various Porcine Tissues after Dehydration

**DOI:** 10.1371/journal.pone.0072573

**Published:** 2013-09-04

**Authors:** Jacob P. Meyer, Kieran E. McAvoy, Jack Jiang

**Affiliations:** University of Wisconsin-Madison School of Medicine and Public Health, Department of Surgery, Division of Otolaryngology – Head and Neck Surgery, Madison, Wisconsin, United States of America; University of California, Irvine, United States of America

## Abstract

The biphasic effects of liquid on tissue biomechanics are well known in cartilage and vocal folds, yet not extensively in other tissue types. Past studies have shown that tissue dehydration significantly impacts biomechanical properties and that rehydration can restore these properties in certain tissue types. However, these studies failed to consider how temporal exposure to dehydrating or rehydrating agents may alter tissue rehydration capacity, as overexposure to dehydration may permanently prevent rehydration to the initial liquid volume. Select porcine tissues were dehydrated until they reached between 100% and 40% of their initial mass. Each sample was allowed to rehydrate for 5 hours in a 0.9% saline solution, and the percent change between the initial and rehydrated mass values was calculated. Spearman correlation tests indicated a greater loss in mass despite rehydration when tissues were previously exposed to greater levels of dehydration. Additionally, Pearson correlation tests indicated the total liquid mass of samples after complete rehydration decreased when previously exposed to higher levels of dehydration. Rehydration rates were found by dehydrating tissues to 40% of their initial mass followed by rehydration in a 0.9% saline solution for 60 minutes, with mass measurements occurring in 15 minute intervals. All tissues rehydrated nonlinearly, most increasing significantly in mass up to 30 minutes after initial soaking. This study suggests the ability for tissues to rehydrate is dependent on the level of initial dehydration exposure. *In vitro* rehydration experiments therefore require controlled dosage and temporal exposure to dehydrating and rehydrating agents to avoid incomplete rehydration, and caution should be taken when combining different tissue types in models of hydration.

## Introduction

Maintaining adequate hydration during *in vitro* tissue experiments is imperative to achieving results that closely mimic physiological *in vivo* conditions. Fluid is a key component in the biomechanical properties of many tissue types. The effects of fluid on tissue biomechanics have been studied in considerable detail, particularly in cartilage and vocal folds where fluid plays a prominent role in defining mechanical behavior. Cartilage [1] and vocal fold [2] properties have been described by constitutive, analytical, and finite element biphasic models that consider the spatiotemporal mechanical contributions of both the solid and fluid contents. Other tissue types, such as tendons and ligaments, also have been investigated with regards to the effects fluid content can have on the tissue mechanics [3], [4]. Knowledge about how fluid alters biomechanical properties in selective tissue types allows for *in vitro* studies to be a more accurate representation of biological conditions.

Because fluid has been found to play significant mechanical roles in such tissues, many previous studies have evaluated how dehydration changes mechanical behavior. For example, Armstrong and Mow [5] and Mow et al. [1] found a direct correlation between decreasing water content and decreasing cartilage permeability, resulting in changes to creep and stress relaxation [6]. In tendons, dehydration reduces strain sensitivity [4] and stress relaxation [7]. In vocal folds, dehydration led to decreased tissue compliance [8]. Dehydration in skin changes tissue stiffness in unpredictable ways [9]. In general there exists a direct relationship between the hydration state of a tissue and its viscoelasticity. This directly impacts creep and stress relaxation, both of which are common rheological measurements for defining tissue properties. While this biphasic effect has been noted in many tissue types, it is likely to play a role in many others.

In addition to dehydration, several previous studies have evaluated the recoverability of tissue mechanics after rehydration of previously dehydrated tissue samples. Chan and Tayama [2] dehydrated vocal fold tissues in sucrose and then rehydrated the tissues in distilled water; the tissues were unable to recover their initial shear moduli and dynamic viscosity values. Similarly, Hanson et al. [10] found that vocal folds were unable to fully rehydrate after sufficient dehydration and thus were mechanically altered despite rehydration. More favorable recovery to initial creep has been observed in ligaments [11], [12] and skin [13].

The rehydration of tissues is an important parameter in the analysis of biphasic properties, and rehydration behavior is inherently variable between tissue types. Unfortunately, previous rehydration studies have typically failed to characterize the extent of fluid change before and after rehydration or have not measured the length of time tissues are exposed to dehydrating and rehydrating agents. Consequently, few studies have considered how rehydration abilities may be affected by temporal exposure to dehydrating or rehydrating agents, and that overexposure to dehydration may permanently prevent rehydration to the initial fluid volume. This relationship was preliminarily posited and observed by Hanson et al. [10] in vocal fold tissue, but was only studied at two levels of dehydration, leading to an incomplete understanding of the rehydration behavior. Given that tissues may be permanently altered with sufficient dehydration, it is imperative that the rehydration capacities of tissues after dehydration be profiled for proper experimentation. In this study we hypothesized that tissues change in their ability to reclaim fluid depending on the extent of initial dehydration. In other words, the amount of fluid loss experienced by a tissue determines the ability of that tissue to regain lost fluid. We predicted that this behavior follows a particular profile, where the original fluid mass cannot be reclaimed by rehydration after experiencing a certain level of dehydration. Furthermore, this level may be dependent on tissue type. Additionally, we hypothesized that tissues rehydrate at a nonlinear rate, and therefore care must be taken to expose tissues to rehydrating agents for a sufficient length of time if the initial fluid contents are to be recovered.

## Materials and Methods

### Tissue Preparation

All tissues were generously donated immediately postmortem by Schmidts Slaughterhouse (Bonduel, WI) from pigs slaughtered for human consumption. Permission was obtained for the use of these tissues in research. Surface lung parenchyma from the right caudal lobe, the tendon of the long digital extensor (prior to branching), elastic cartilage from the ear, abdominal dermis with epidermis, abdominal fat, and gastrocnemius muscle tissues were harvested immediately postmortem from the same animal. Tissues were then sectioned into approximately 1.5 cm×1.5 cm×0.5 cm pieces using microsurgical tools, and care was taken to avoid deforming the tissue to prevent expulsion of internal fluids. Tissues were placed in a 0.9% saline solution with density of 1.004 g/mL, frozen, and stored at −12°C. A previous study has shown that storing soft tissue in freezing conditions does not significantly affect their viscoelastic properties [14], and therefore is not thought to alter the solid matrix interactions with water or internal water properties of the samples after thawing. Frozen tissue samples were thawed in a water bath at room temperature (25°C) immediately prior to each experiment and kept in 0.9% saline up until dehydration.

### Tissue Dehydration and Rehydration

Twenty-four prepared tissue samples were randomly selected from each tissue type. Each individual sample was dabbed with a cloth to remove excess surface fluid and weighed on an analytical electronic balance (Mettler Toledo, Columbus, OH). The initial recorded mass of each sample (*M_T_*) was considered to be the total mass of both the liquid and solid constituents of the tissue sample. Dehydration was performed by placing the tissues into a vacuum oven (Fisher Scientific, Pittsburgh, PA) at 38°C. This method was previously used to simulate homogeneous and systemic dehydration throughout the tissue structure as described by Hanson et al. [10]. Tissue samples were selectively dehydrated until they measured between 100% and 40% of *M_T_* (i.e. after a 0%–60% change in mass). This new mass was recorded as *M_D_*. Although this range of dehydration includes values well beyond those typically experienced physiologically, it serves to demonstrate possible consequences of dehydrating tissues without close quantitative observation. In addition, this range provides comparisons to hypotheses presented by Hanson et al. [10].

**Figure 1:**
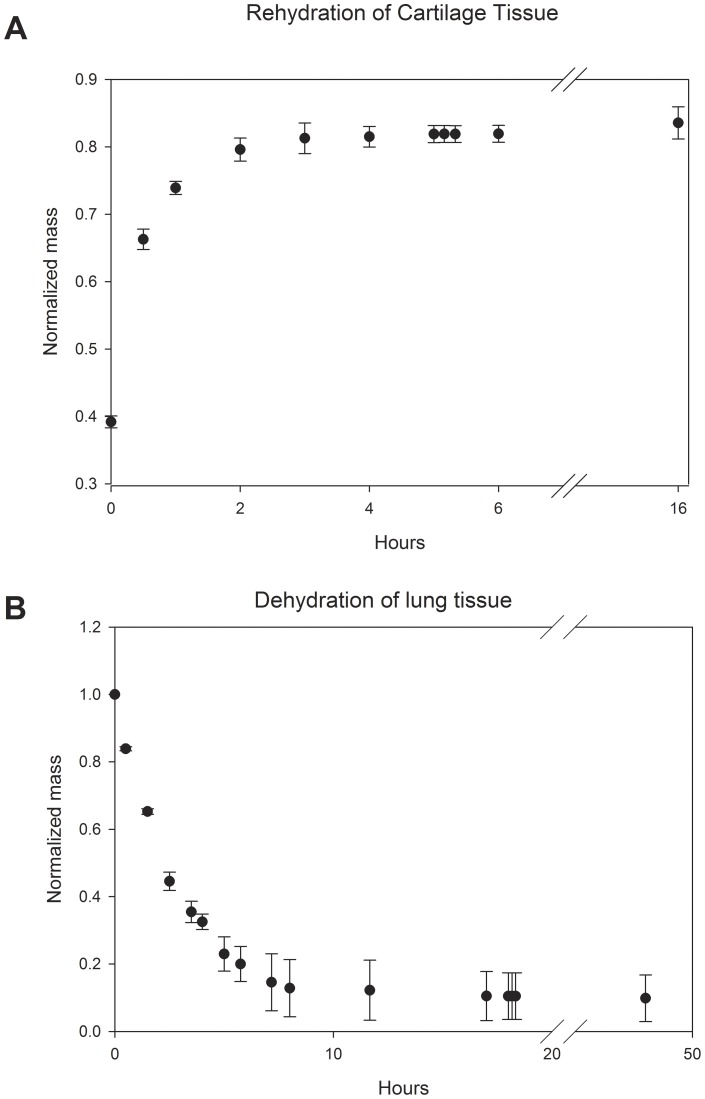
1A: Normalized mass measurements of previously dehydrated cartilage tissues (n = 4 samples) as a function of rehydration time. Tissues were dehydrated to 40% of their initial mass and subsequently placed in a 0.9% saline solution. Mass was measured every hour for 6 hours, with 3 consecutive measurements occurring at the 5th hour in 10 minute intervals. A final measurement was recorded at the 16^th^ hour after rehydration. Mass appeared to be steady after 5 hours, and was not statistically different from that after 16 hours. 1B: Normalized mass measurements of lung tissue as a function of dehydration time (n = 4). Tissues were placed in a 38°C oven then weighed approximately every hour for the first 8 hours. Measurements were then taken at approximately the 12th, 17th, 18th, 18.5, and 48^th^ hour after dehydration. Mass appeared to be steady after 18 hours, and was not statistically different from that at 48 hours. Bars indicate ± standard deviation Results for (a) and (b) are representative of all tissues used in this study.

After dehydration, each sample was placed in 0.9% saline solution for 5 hours in order to provide ample rehydration time as determined by preliminary studies (Figure 1A). After 5 hours, each sample was dabbed and successively weighed 3 times in 10-minute intervals, being placed back in the saline solution between measurements. A constant weight over this period indicated the samples were saturated with fluid and would not absorb more within a reasonable length of time, and was recorded as *M_R_*. We did not observe any cases in which this requirement was not met after 5 hours. The saline solution was chosen in accordance with a previous study by Hanson et al. [10] to approximate the tonicity of physiologic fluid, and is similar to the phosphate buffered saline (PBS) often used in hydration experiments [7]. After rehydration in saline, the tissues were placed back into the 38°C oven and allowed to dehydrate for 18 hours. Afterwards, tissues were weighed in 3 successive 10-minute intervals to ensure a steady mass as before. This mass was used as the solid mass *M_S_*, as preliminary work indicated a steady mass at this time was indicative of the final solid mass (Figure 1B). The solid mass was assumed to remain independent of the hydration state of a tissue sample, and was used to calculate the liquid mass by subtracting it from the total mass. As a result, the liquid mass fraction of a sample (i.e. the fraction of a sample's mass attributed to liquid) prior to dehydration was calculated by,
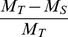
(1)and the liquid mass fraction after rehydration was calculated by substituting *M_R_* for *M_T_* into Equation 1.

### Tissue Rehydration Rate

In addition to calculating the total rehydration capacity of each tissue type, the rate of rehydration was also investigated. Four samples from each of the six tissue types were dehydrated to 60% of their respective initial masses using the method described earlier. The samples were then placed in 0.9% saline solution for 60 minutes, during which the masses were measured at 15 minute intervals. As before, the tissues were dabbed immediately prior to mass measurements.

### Data and Statistical Analysis

Data were analyzed and graphed using SigmaPlot v11.0 (SystatSoftware, Inc., San Jose, CA) and MATLAB (The Mathworks, Natick, MA). A Spearman's rank correlation analysis was performed to determine the statistical dependence between the percent changes in mass after rehydration compared to the percent changes in mass after dehydration. An alpha less than 0.05 and Spearman's correlation constant greater than 0.5 or less than −0.5 were considered significant for dependency. *T*-tests were also used to compare means of masses within and between tissue groups.

## Results

**Figure 2:**
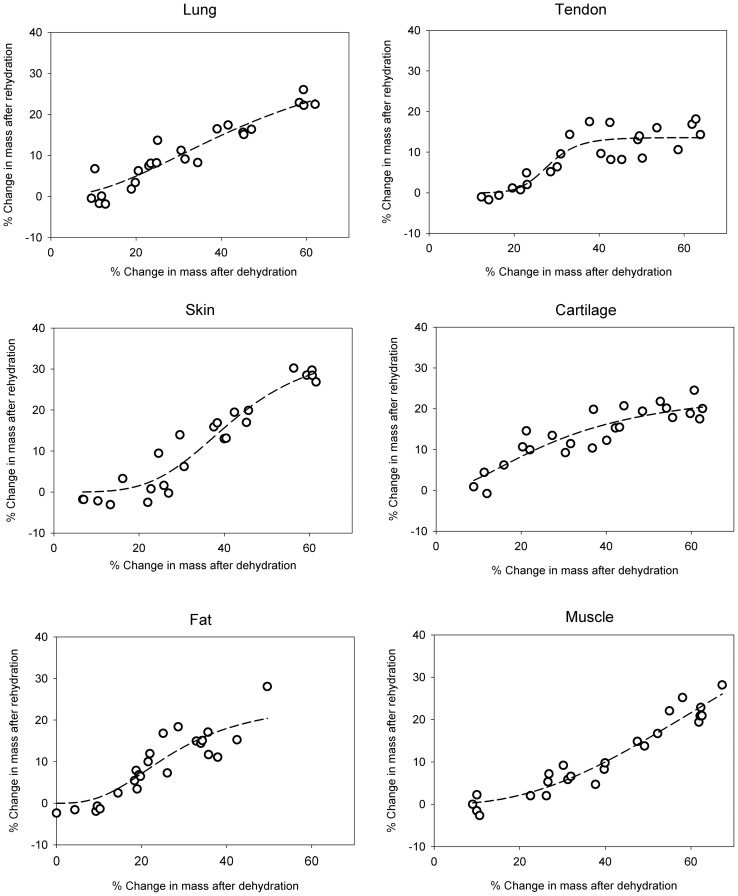
Percent change in mass for each tissue type after rehydration as a function of percent mass after dehydration. Positive percentage changes correspond to loss in fluid mass compared with the initial masses, and negative percent changes indicate absorbance of fluid greater than the initial fluid volume. Tissues nonlinearly tend to experience a greater change in mass after rehydration when previously exposed to larger levels of dehydration. N = 24 for each tissue.

The resulting dependence of rehydration capacity on the percent change in mass after dehydration (0%–60%) is shown in Figure 2. Data are presented as the percent change in the original mass after rehydration 
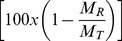
 as a function of the percent change in mass after dehydration 
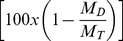
. A low percent change after rehydration indicated that the tissue was able to recover most of the initial fluid volume lost during dehydration. Similarly, negative percent changes after rehydration indicated that tissue samples absorbed more than the initial amount of fluid. Table 1 shows the Spearman correlation constants and respective p-values for the percent change in mass after rehydration compared to the percent change in mass after dehydration within each tissue type. All of the tissues displayed a pattern in which higher levels of dehydration were nonlinearly correlated to higher losses in mass after rehydration. Therefore it appears that the re-absorption capacity of a tissue is related to the amount of dehydration previously experienced by it.

**Table 1:** Spearman correlation constants and p-values for percent change in mass after rehydration compared to percent change in mass after dehydration.

	Lung	Tendon	Skin	Cartilage	Fat	Muscle
Spearman Correlation Constant	0.946	0.844	0.924	0.868	0.504	0.945
p-value	<0.01	<0.01	<0.01	<0.01	0.012	<0.01

**Figure 3:**
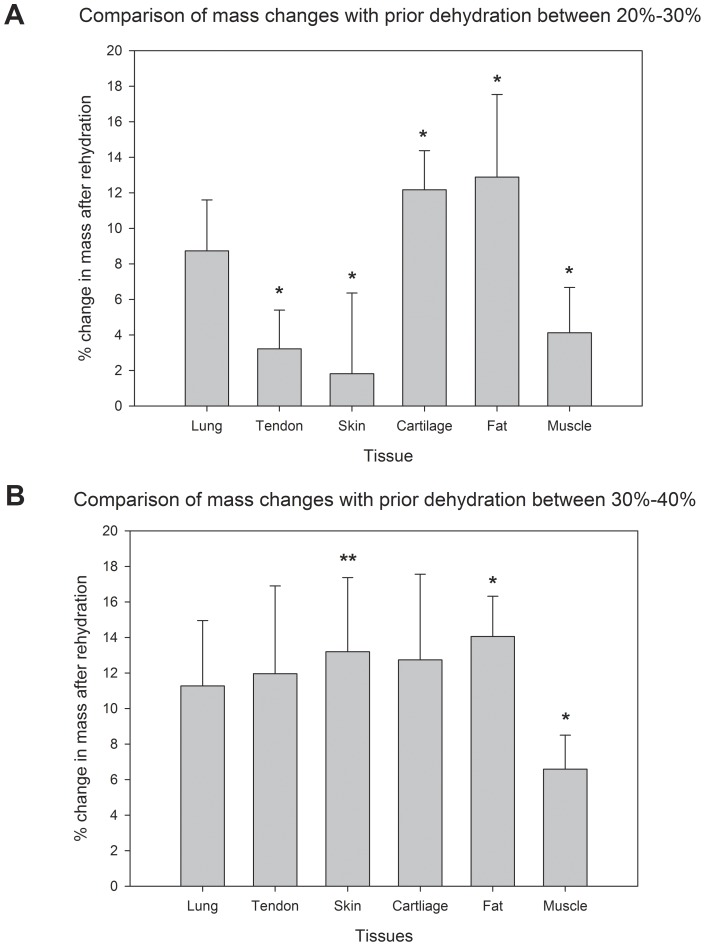
3A: Percent change in mass after rehydration for tissue samples previously exposed to 20%–30% dehydration. Cartilage and fat tissues had significantly greater changes in mass compared to tendon, skin, and muscle. Lung tissue was not statistically different from any other. (tendon, cartilage, muscle: n = 4; lung, skin, fat: n = 5). 3B: Percent change in mass after rehydration for tissue samples previously exposed to 30%–40% dehydration. Skin and fat tissues had significantly greater changes in mass compared to muscle, but all other tissues had no difference. Bars indicate ± standard deviation (lung, tendon, cartilage, muscle: n = 4; skin: n = 5; fat: n = 6). *p<0.01, **p<0.05.

Differences between the data trends were also analyzed, particularly at the 20%–30% and 30%–40% dehydration intervals where the trends were most unique as illustrated in Figure 2. Figure 3 depicts mean changes in mass after rehydration grouped by dehydration levels between 20%–30% (Figure 3A) and 30%–40% (Figure 3B). At the lower dehydration interval, both cartilage and fat samples experienced a significantly greater loss in mass after rehydration than did tendon, skin, and muscle (p<0.01). However, at the 30%–40% interval, only muscle experienced a significantly lower change in mass after rehydration compared to skin (p<0.05) and fat (p<0.01). Consequently, several of the tissues responded differently to prior dehydration, and muscle was capable of recovering from higher levels of dehydration than the other tissues.

**Figure 4:**
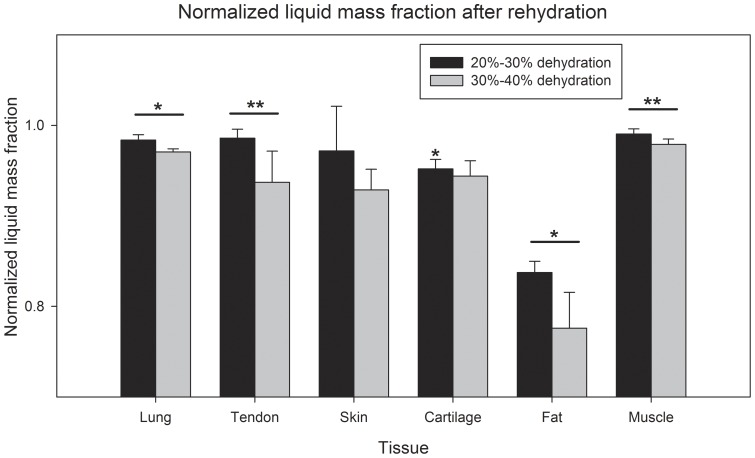
Normalized liquid mass fractions of tissue samples after rehydration compared to 20%–30% or 30%–40% change in mass incurred by dehydration. The liquid mass fraction after rehydration significantly decreased for lung, tendon, fat, and muscle when exposed to 30%–40% dehydration. Bars indicate ± standard deviation. For 20%–30%: tendon, cartilage, muscle: n = 4; lung, skin, fat: n = 5. For 30%–40%: lung, tendon, cartilage, muscle: n = 4; skin: n = 5; fat: n = 6. *p<0.01, **p<0.05.

In addition to the percent changes in total mass, the solid mass (*M_S_*) was used to calculate and compare the liquid mass fractions of tissues. Figure 4 shows mean values for liquid mass fractions after rehydration grouped by prior dehydration level. Samples within the 20%–30% and 30%–40% dehydration ranges were analyzed as in Figure 3. The liquid mass fractions found after rehydration were normalized to the initial values prior to dehydration for each sample, and therefore accounted for differences within and between tissue types. Although all of the mean mass fractions calculated were found to decrease in the 30%–40% group from the 20%–30% group, only lung (p<0.01), tendon (p<0.05), fat (<0.01) and muscle (p<0.05) tissues had a significant change.

**Figure 5:**
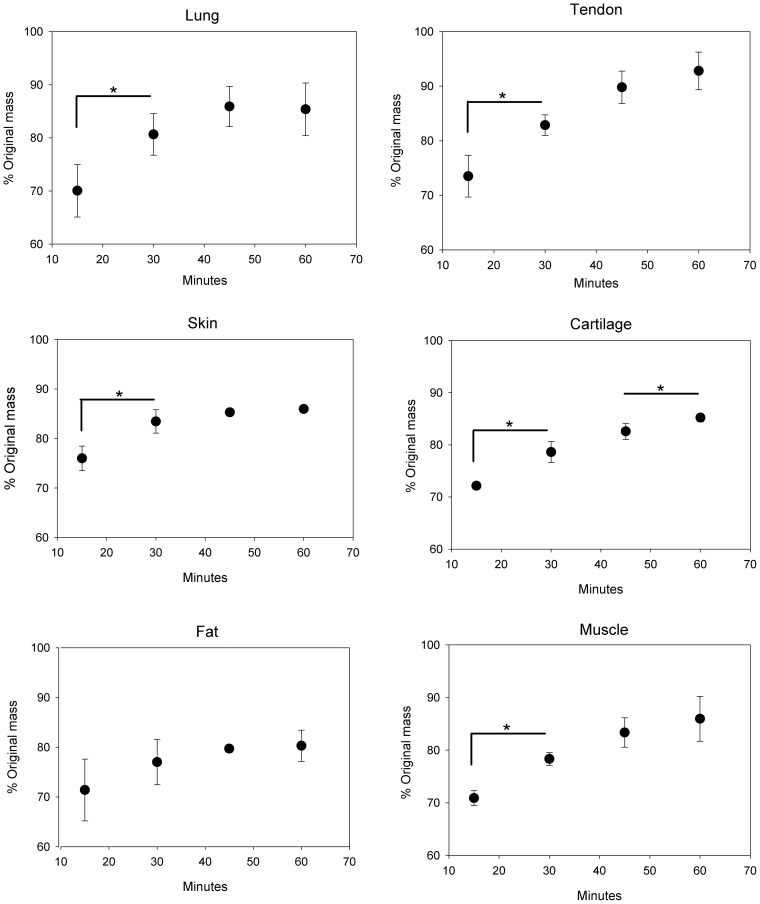
Percent of the original mass of each tissue type after rehydrating for 15, 30, 45, and 60 minutes in 0.9% saline solution. All tissues began at 60% of their original mass. N = 4 for each tissue. Bars indicate ± standard deviation. *p<0.05.

The results from the rehydration rate tests are shown in Figure 5, where the increase in mass due to fluid reclamation after a 40% reduction in mass from dehydration is shown as a function of time. The mean (± standard deviation) mass values as a percent of the original mass prior to dehydration for each data point depicted in the figure are given in Table 2. All of the tissues absorbed fluid nonlinearly over time and, except for fat tissue, had significant (p<0.05) increases in mass after 30 minutes of soaking. Furthermore, cartilage tissues experienced a significant (p<0.05) increase in mass even after 60 minutes of soaking.

**Table 2:** Mean (± standard deviation) percent of original tissue mass during rehydration (n = 4 for each tissue). All tissues started at 60% of the original mass.

Tissue	15 minutes	30 minutes	45 minutes	60 minutes
Lung	70.04±4.93	80.64±3.93	85.89±7.76	85.37±6.96
Tendon	73.49±3.83	82.84±1.90	89.78±2.98	92.78±3.45
Skin	75.99±2.49	83.46±2.38	85.28±0.56	85.96±0.74
Cartilage	72.14±0.59	78.61±2.01	82.56±1.56	85.20±0.77
Fat	71.40±6.21	77.01±4.56	79.71±0.76	80.29±3.15
Muscle	70.89±1.44	78.33±1.21	83.36±2.80	85.95±4.27

## Discussion

The results from rehydrating different tissues suggest that the initial level of dehydration plays a role in the ability of tissues to rehydrate. Rehydration capacity appears to be correlated to the initial dehydration level of tissues. Additionally, significant decreases in the liquid mass fraction after complete rehydration were found in lung, tendon, fat, and muscle tissue when previously experiencing a 30%–40% loss in mass from dehydration. Together, these results suggest that it is necessary to monitor the change in mass during dehydration if the tissue is to be rehydrated.

Interestingly, every tissue studied had the capacity to fully recover its liquid mass after experiencing some degree of dehydration, leading to a toe region with a shallow slope (Figure 2). In addition to the toe region found at low levels of dehydration, the change in mass after rehydration flattened out after a certain level of dehydration for several of the tissues. This was an interesting behavior in that increasing levels of dehydration had a decreasing effect on the percent change in mass after reaching a certain inflection point. This property was particularly pronounced in tendon, but a notable exception was muscle tissue, which did not exhibit this behavior within the levels of dehydration studied. Between this region at high levels of dehydration and the toe region at low levels of rehydration, most of the tissues had a transitory period of dehydration in which the percent change in mass after rehydration was primarily affected.

The amount of dehydration that was tolerated before reaching this transitory region, however, seemed to be dependent on tissue type. In particular, fat and cartilage tissues had shortened toe regions and exhibited significantly greater mass changes after rehydration when exposed to 20%–30% dehydration (Figure 3). Lung tissues behaved similarly, but were not found to have significantly different losses than the other tissues. Even so, such differences between recovered masses were mostly eliminated once tissues were dehydrated by 30%–40%. At this level, most tissues, except muscle, exhibited similar losses in mass upon rehydration. These results indicated that each tissue type responded differently to prior dehydration, likely the result of differing tissue content. It is therefore necessary to characterize the rehydration tendencies for a particular tissue type when experimenting with rehydration dynamics. For example, the general hydrophobic nature of fat tissue may have affected significant fluid re-absorption and played a role in limiting this ability. This manifested as a sensitivity to smaller amounts of dehydration otherwise tolerated by other tissues. Similarly, cartilage was found to steadily lose the ability to fully recover fluid over increasing levels of dehydration. Although cartilage is well known for its ability to sequester liquid, we speculated that the stiffness of the samples we observed may have prevented re-swelling of the structure upon rehydration. The precise cause for this, however, remains unknown and should be studied further. Nevertheless, studies that use rehydrated cartilage must closely monitor the dehydration levels, as dehydration quickly affects the final mass restored upon rehydration.

We also investigated the time dependency of rehydration for each tissue type. Although the exact rates of rehydration varied, all tissues re-absorbed fluid nonlinearly as anticipated. Tissues absorbed fluid at rates that gradually decreased over time, until the rate reached an asymptote and all recoverable fluid was absorbed. Every tissue with the exception of fat demonstrated a significant increase in mass after 15 and 30 minutes of rehydration. After 45 minutes, mass increases fell below significant in all tissues except cartilage, which even after 60 minutes showed a statistically significant increase in mass. A lack of a significant increase in mass, however, did not necessarily mean the tissues were fully rehydrated, but rather may have slowed down and required longer intervals of time before acquiring significant increases.

These results illustrated that dehydrated tissue samples must be given adequate time to rehydrate after being dehydrated, and that this time varies according to tissue type due to differences in microstructure. While the length of time required to rehydrate is likely to vary depending on the method of rehydration, it is evident that this must be determined prior to experimentation with rehydrated tissues, and tissues cannot be rehydrated for arbitrary lengths of time. It is also possible that the rehydration rate may vary according to the amount of initial dehydration. Any microstructual changes resulting from large dehydration levels could have a significant impact on the rate. Therefore, future studies should focus more on how the extent of initial dehydration can alter the rehydration rate.

The differences in rehydration profiles and rates between tissues may be dependent on many factors, including extracellular matrix components and microstructure. These differences limit our ability to make clear distinctions between properties that may or may not affect hydration behavior. The method by which tissues were prepared for this study may also limit the precision of the results, as the tissues likely incurred microstructural damage from lacerations during isolation as well as during the freezing and thawing processes. Similarly, the methods of this study were not exhaustive, and different rehydration rates may be observed if using other rehydration methods. It is possible that using distilled water instead of saline would recover more fluid after greater levels of dehydration due to its hypotonic state. Future studies should be done to compare the effects of different solutions on rehydration rates and capacities. Likewise, the saline solution used for hydration did not perfectly match the tonicity within the tissues, as fluid absorption above the initial mass was observed in tissues below 10% dehydration (indicated by a negative percent change in mass after rehydration in Figure 2). Although we expected some negative values from inconsistencies in mass measurements due to residual surface liquid, this was especially prominent in skin tissue and may have been reflective of different tonicities in the tissue or microstructure. Such analysis, however, was beyond the scope of this work.

Despite these limitations, this study demonstrates the importance of measuring the extent of dehydration when experimenting *in vitro* with tissue samples. For example, Vargas et al. [13] found that skin only partially recovered properties after rehydration. However, the amount of time that the tissue samples were allowed to undergo dehydration or rehydration was not recorded. Therefore, it is unknown if the results were due to the extent of dehydration or inadequate rehydration time. Our results shed light on the importance of using tissue rehydration profiles to control for such confounding variables in future experimentation. This also has implications for computational models created to predict tissue properties. Models involving the hydration states of tissues can use the results from this work to form a basis for correctly accounting for changes in rehydration capacities and rates of hydration. Similarly, our results illustrate the pitfalls of lumping different soft tissues together when modeling hydration states because hydration behaviors can vary between tissue types. Future studies should directly measure how dehydration affects microstructure organization in tissues, and how these effects may alter mechanical properties. In particular, biphasic-related measurements, such as creep, relaxation, and permeability should be investigated. These studies would help to determine whether a biphasic effect (behavior dependent on both solid and liquid phases) is occurring in other tissue types that have not been previously noted, as well as support biphasic understandings in tissues such as cartilage.
